# Soft repression: Subtle transcriptional regulation with global impact

**DOI:** 10.1002/bies.202000231

**Published:** 2020-11-20

**Authors:** Anindita Mitra, Ana-Maria Raicu, Stephanie L. Hickey, Lori A. Pile, David N. Arnosti

**Affiliations:** 1 Department of Biological Sciences, Wayne State University, Detroit, Michigan, USA; 2 Cell and Molecular Biology Program, Michigan State University, East Lansing, Michigan, USA; 3 Department of Computational Mathematics, Science, and Engineering, Michigan State University, East Lansing, Michigan, USA; 4 Department of Biochemistry and Molecular Biology, Michigan State University, East Lansing, Michigan, USA

**Keywords:** gene regulation, metabolism, promoter, repression, retinoblastoma, SIN3, transcription

## Abstract

Pleiotropically acting eukaryotic corepressors such as retinoblastoma and SIN3 have been found to physically interact with many widely expressed “housekeeping” genes. Evidence suggests that their roles at these loci are not to provide binary on/off switches, as is observed at many highly cell-type specific genes, but rather to serve as governors, directly modulating expression within certain bounds, while not shutting down gene expression. This sort of regulation is challenging to study, as the differential expression levels can be small. We hypothesize that depending on context, corepressors mediate “soft repression,” attenuating expression in a less dramatic but physiologically appropriate manner. Emerging data indicate that such regulation is a pervasive characteristic of most eukaryotic systems, and may reflect the mechanistic differences between repressor action at promoter and enhancer locations. Soft repression may represent an essential component of the cybernetic systems underlying metabolic adaptations, enabling modest but critical adjustments on a continual basis.

## INTRODUCTION

The first transcriptional regulation characterized in bacterial systems involves repressors described to function as on/off switches. Indeed, phage lambda repressor delivers tight repression to maintain lysogeny, while the LacI repressor can silence an otherwise highly transcribed operon, depending on nutritional status. Interestingly, subsequent studies have shown that *lacZ* expression can be delicately tuned over a wide range, depending on graded input from activators and repressors.^[1]^ In eukaryotes, transcriptional repression reflects input from a wider set of regulators: inherent chromatin barriers, histone modifications that facilitate heterochromatin formation, and combined action of DNA-binding transcription factors and corepressors that they recruit, including the evolutionarily conserved retinoblastoma (Rb) and SIN3 family proteins.

The action of transcriptional repressors and corepressors has played a central role in many studies of developmental biology, where such proteins are essential mediators of tissue-specific gene expression, as well as controllers of cell cycle, and circadian regulation.^[2–4]^ Inducible gene expression required for physiological response to environmental fluctuations also involves the deactivation of repression complexes, for instance in the upregulation of stress-responsive genes.^[5]^ In many systems, the effectiveness of repression is essentially complete, and depends on reaching a critical concentration of relevant transcription factors, or intensity of signaling pathways that permit the assembly (or disassembly) of repression complexes. To achieve a cell-type specific response, target genes are repressed below some threshold that ensures establishment and/or maintenance of a specific cell state. For instance, Blimp-1/PRDM1 is a tissue-specific repressor whose key role is in driving plasma cell differentiation and silencing genes involved in immune response.^[6,7]^ Its loss in maternal uterine tissue has been shown to upregulate hundreds of genes that are normally silenced.^[8]^ Likewise, the RE-1 silencing transcription factor (REST) is a regulator of cell differentiation. REST is ubiquitously expressed in non-neuronal cells for the silencing of neuronal genes, while mostly absent from differentiated neurons.^[9]^ Its loss in quiescent neural progenitors leads to neural differentiation, suggesting its role is to prevent neural differentiation through gene silencing.^[10]^ In contrast to this choice between silencing or activity, molecular genetic studies have identified numerous genomic targets of repression complexes that may be less dramatically impacted by the presence of such regulatory factors. Metazoan transcription factors and their corepressors are typically found to physically interact with thousands of genes, yet perturbation experiments frequently show only a small subset with significantly altered expression. This disparity is usually ascribed to some degree of “off target” interactions, whereby these complexes do not have a significant function at some loci.^[11]^ An additional possibility is that there are context-dependent interactions, in which the binding to some genes may be essential for regulation only in certain cell types, or under specific conditions that may not have been assessed in a particular experiment.

A nonexclusive, alternative explanation to the presence of certain physical repressor complex interactions is that the type of repression that is biologically significant is of a form that is inherently “soft,” that is, altering expression, but not in an absolute on/off fashion. Such regulation may be especially important for widely-expressed “housekeeping” genes, where expression rarely, if ever, is silenced. As we have argued with respect to Rb corepressors, binding and coordinate regulation of ribosomal protein genes may represent just such a case.^[12]^ A second example is that of regulation of genes in methionine catabolism by the SIN3 cofactor, where perturbation to SIN3 levels induces approximately two-fold changes in relevant pathway genes.^[13]^ Significant for the analysis of such datasets, the amount of repression may be subtle, and in some cases, less than the extent typically required to clearly differentiate signal from noise.

Here, we discuss studies of Rb and SIN3, two essential and conserved corepressor protein families, providing a picture of the diverse targets with which these transcriptional regulators are physically and functionally associated. We propose that soft repression is a major contributor to gene regulatory control and plays a key role in metabolic adaptation. The unique soft transcriptional responses of some genes to corepressor regulation may result from the complexity of signaling at the respective promoters, or from the different effects of repressor complexes acting at promoters versus enhancers. Importantly, we suggest that the action of these corepressors may represent a wider, unappreciated phenomenon impacting a great number of eukaryotic regulatory factors and pathways. Further investigation will uncover the significance of this second, less dramatic form of transcriptional regulation.

### The hypothesis formalized:

Canonical models for the action of transcriptional repressor proteins often emphasize the possibilities for tight control through on/off action, enabling exquisite tissue-specificity and physiological control. A number of genomic studies, however, have increasingly pointed to a pleiotropic “soft repression” mechanism of action on widely expressed genes, whose modulation may be subtle. Using two well-studied corepressor families, Retinoblastoma and SIN3, we hypothesize that some promoter proximal corepressors function to modestly attenuate gene expression in a biologically meaningful way-a mechanism that may be especially prominent on genes featuring multiple regulatory inputs.

### Testing the hypothesis:

To better understand soft repression, we propose the application of diverse technologies: (1) using high throughput RNA-sequencing methods with deeper sequencing and greater number of biological replicates to increase resolution and discern the difference between noise and soft repression; (2) single cell transcriptomic studies, which will circumvent discrepancies that might arise from heterogeneous cell populations; (3) nuclear run-on-based technologies such as GRO-seq, to allow for the assessment of immediate transcriptional impacts of corepressor perturbation; (4) targeting the corepressor directly to single gene promoters to perturb a specific circuit and avoid pleiotropic effects from global manipulations of the repressor; (5) a thorough computational consideration of soft-repression in interpreting population- and species-level cis-regulatory variation. We urge gene expression researchers to consider soft repression as a significant and biologically relevant form of transcriptional regulation in future studies and test the hypothesis using these proposed methods.

## THE RETINOBLASTOMA TUMOR SUPPRESSOR PROTEIN MEDIATES BOTH HARD AND SOFT REPRESSION

The retinoblastoma tumor suppressor protein (Rb) is a conserved transcriptional corepressor present in most eukaryotes including plants, animals, and microbes. The study of the *RB1* gene stems from research by Knudson, who linked mutations in the gene to retinoblastoma, an eye cancer presenting in early childhood.^[14]^ Since then, its role in cancer, development, and normal physiology has been extensively studied in a variety of systems. Most eukaryotes express a single Rb protein, but the gene has been duplicated in select lineages including in vertebrates and Drosophila. In humans, the Rb family comprises Rb, p107, and p130, which exhibit partially overlapping as well as non-redundant functions in development and cancer.^[15]^ Similarly in Drosophila, paralogs Rbf1 and Rbf2 represent an ancient duplication event, where Rbf1 appears to have more roles in cell cycle regulation, while Rbf2 may interact with and regulate an extensive set of genes linked to growth control and metabolism, including ribosomal protein genes.^[16]^ Below, we summarize basic properties of these proteins with a focus on work from Drosophila; vertebrate paralogs of Rb have been similarly examined in countless studies in the context of development and disease.

Rb proteins regulate genes by binding to E2F family transcription factors found on promoters. E2F factors have a canonical role in the regulation of cell cycle genes that are transiently induced during the cell cycle. In Drosophila G1, Rb binds to the E2F-DP heterodimer and blocks E2F from activating expression of downstream genes such as *cycA, cdc2,* and *DNApola,* which are required for S phase entry.^[17]^ Rb-mediated repression is relieved later in G1 as Rb is inhibited via phosphorylation, and the cell enters S phase. Similar regulation appears to apply to promoters active later in the cell cycle, such as *cycB.* This cell-cycle regulatory role by Rb proteins is highly conserved in eukaryotes.^[18]^ Initial characterization of Rb function derived from its cancer-associated phenotype, and led to cell cycle regulation as a central area of study. However, genome-level studies soon revealed a plethora of other regulatory roles.

**FIGURE 1:**
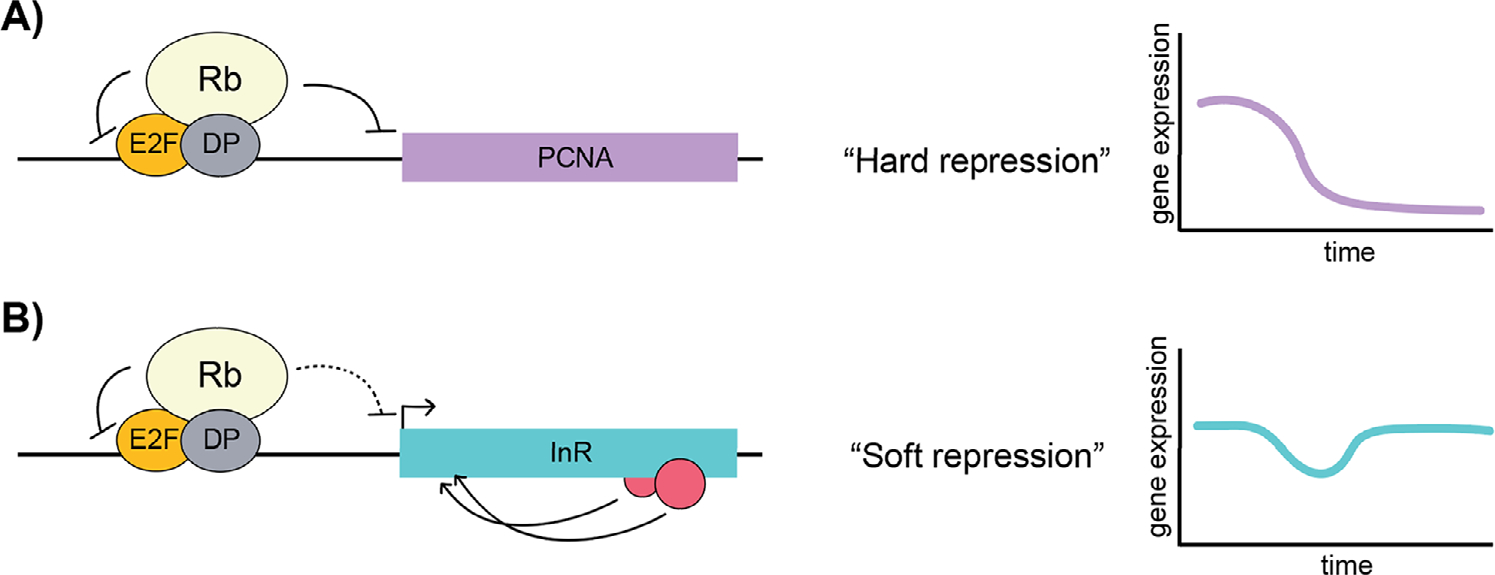
Comparison of hard versus soft repression. (A) Rb can function as a potent repressor on certain genes such as the cell cycle-related *PCNA* by blocking the E2F transactivation domain and inducing a repressed chromatin state. (B) In contrast, on other genes such as *InR,* Rb functions in concert with other factors that may have to balance each other’s activities, leading to more moderate repression of the gene

Transcriptomics studies have uncovered diverse classes of genes that are differentially expressed after Rb loss or overexpression. Pioneering studies using cultured Drosophila S2 cells showed that Rbf1 knockdown affected canonical cell cycle, DNA replication, and DNA repair genes, but also a host of non-cell cycle-related genes.^[19]^ Interestingly, specific promoters tested in transfection assays were differentially sensitive to the Rb paralogs - most, but not all, cell cycle genes being more sensitive to repression by Rbf1.^[16,20,21]^ In contrast, Rbf2 has preferential action on certain ribosomal protein promoters, with which it is prominently associated in vivo, although the extent of regulation is much more modest than that seen for cell cycle genes.^[12,16]^ Knockdown studies in human fibroblasts similarly illustrate that loss of each human Rb family member misregulates diverse classes of genes.^[22]^ Rb knockdown led to upregulation of DNA replication, DNA metabolism, and cell cycle genes; p107 knockdown led to downregulation of genes involved in oxidative phosphorylation, electron transport, and NADH dehydrogenase activity; genes involved with organelles were upregulated after p130 knockdown. Although the regulation was not shown to be direct in all cases, these data indicate unique roles of Rb proteins related to cell metabolic processes. More recently, Rb loss was implicated in reprogramming of glucose tolerance, oxidative metabolism, glutathione synthesis, glutamine catabolism, and nucleotide metabolism in Drosophila.^[23]^ We, and others, have found that the extent to which these target genes are repressed by Rb varies. From cell culture assays performed in our laboratory, we suggest that significant and potent decreases in expression of certain genes such *PCNA* represent hard repression, in which the gene is turned off for a period of time, while more moderate decreases observed, as is the case with *InR,* represent what we term soft repression (Figure 1).

Overall, comparison of genes directly bound by Rb family proteins in diverse organisms suggests that at least a portion of targeting is widely conserved, extending beyond the canonical cell cycle category. For instance, the human p130 protein is especially enriched on mitochondrial and cytoplasmic ribosomal protein promoters, similar to the pattern for Rbf1/2 in Drosophila, suggesting that this class of genes may represent a common target.^[12,22]^ While Rb family members are typically found proximal to the transcriptional start site, human Rb proteins have also been found to localize to gene enhancers, and DNA repeat elements like LINEs and SINEs, where they recruit cofactors to change histone marks.^[24,25]^ Still, Rb proteins are preferentially localized to promoter proximal regions, perhaps due to reliance on recruitment by E2F transcription factors, which are also generally located near the transcriptional start site.^[12]^ Overall, emerging research indicates that retinoblastoma proteins exhibit highly conserved functional roles, which may include both hard and soft repression on distinct targets.

### THE SIN3 COREPRESSOR REGULATES GENES INVOLVED IN ESSENTIAL CELLULAR PROCESSES

SIN3 is a broadly acting transcriptional regulator conserved from yeast to humans. Pioneering genetic studies identified this factor as a key regulator of mating type switching in *Saccharomyces cerevisiae*.^[26,27]^ The SIN3 protein itself does not possess enzymatic activity but rather acts as a scaffold that interacts with other factors including histone deacetylases and histone demethylases.^[28]^ Some eukaryotes, such as budding yeast and *C. elegans,* possess a single *SIN3* gene, as does Drosophila (*Sin3A*), where alternative splicing produces multiple protein isoforms. Diversity is achieved in *Schizosaccharomyces pombe* and vertebrate species through multiple SIN3 paralogs, including the mammalian *SIN3A* and *SIN3B* genes. Mutations and altered expression of these mammalian paralogs are found in diseases such as breast cancer, pancreatic cancer, and Witteveen-Kolk syndrome, a neurodegenerative disorder.^[29–31]^

Early genome-wide transcriptomic analyses from multiple species revealed that SIN3 acts both as a corepressor and as a coactivator of transcription.^[32–34]^ SIN3 was found to regulate genes involved in many different cellular processes, including cell cycle, metabolism, DNA replication, and stress response. Regulatory roles of the factor are evolutionarily conserved; for instance, SIN3 regulates cell cycle progression in organisms from yeast to mammals. In Drosophila S2 cells, reduction of *Sin3A* results in a change in expression of a number of cell cycle regulators, including a decrease in the level of *string,* required for the G2 to M transition, which halts the progression of cell cycle.^[33,35]^ This same step in the cell cycle is affected by knockout of *mSin3a* in mouse embryonic fibroblasts.^[34,36]^ The SIN3B paralog is not required for cell proliferation but rather interacts with the DREAM complex to repress essential genes and enable maintenance of quiescence.^[37,38]^

Studies from Drosophila show that genes of housekeeping pathways are also bound and regulated by Sin3A. Sin3A influences mitochondrial function by regulating the expression of multiple nuclear-encoded mitochondrial genes that encode electron transport chain subunits.^[33,39]^ By regulating expression of mitochondrial genes, as well as genes and metabolites of the glutathione pathway, Sin3A influences overall fitness and response to oxidative stress.^[33,40,41]^ Expression of enzymes involved in energy production are also regulated by the Sin3A complex.^[33,39]^ *Sin3A* knockdown in Drosophila S2 cells affects the gene expression and metabolite levels of several glycolytic and TCA cycle intermediates.^[33,41]^ The reduction of Sin3A also affects expression of genes encoding enzymes of methionine metabolism and leads to a decrease in levels of the methyl donor *S*-adenosylmethionine (SAM).^[13]^ These findings indicate that an important function of the Sin3A complex is to regulate expression of genes encoding enzymes of metabolic pathways to maintain cellular homeostasis.

SIN3 also regulates response to stress in both fly and mammalian models.^[40,42]^ The knockdown of *Sin3A* in Drosophila leads to reduction in expression of genes encoding proteins required for glutathione synthesis as well as increased susceptibility to oxidative stress, a sensitivity that is rescued by glutathione supplementation.^[40]^ A study in mammalian cancer cell lines showed that SIN3B is important in the stress response to treatment with different DNA-damaging agents.^[42]^ Following treatment, there is an increase in expression of SIN3B at the transcript and protein level, which is p53-dependent. Additionally, when *Sin3B* is knocked down during damage, p53 target stress response genes are affected, linking SIN3B to the p53-mediated response to DNA damage.

As noted for Rb family proteins, ChIP studies from worms, flies, and mice show that SIN3 is generally localized to promoter proximal sequences of target genes, and not at distal enhancers.^[43–45]^ Consistent with this pattern, SIN3 and other components of the complex immunoprecipitate with H3K4me3, a promoter-associated mark.^[46]^ In addition to recruitment to the promoter, SIN3 has been reported, in mouse and yeast cells, to localize to gene bodies of some active genes through association with a complex of proteins distinct from those found at promoters. The Rpd3S complex in yeast, which contains Sin3, the Rpd3 HDAC, and two additional factors, is recruited through interactions of complex subunits with histone H3K36me3.^[47,48]^ The predominant role of Rpd3S is to facilitate deacetylation of nucleosomes after the passage of RNA polymerase II, suppressing cryptic initiation along the gene body.^[47,48]^ In the mouse, the Sin3B isoform, along with HDAC1 and homologs of the other two yeast Rpd3S complex members, is found enriched at sites downstream of the promoter of select housekeeping genes.^[49]^ Knockdown of Sin3B leads to an increase in RNA polymerase II levels in the gene body and approximately two-fold activation of GAPDH and RPL13α expression. The mechanism of action of SIN3 in gene regulation along gene bodies is likely distinct from that of SIN3 localized to the promoter proximal region. As a scaffold, SIN3 interacts with a number of protein partners. Localization is likely to reflect recruitment by different sequence-specific DNA binding transcription factors and through recognition of specific combinations of histone modifications by chromatin binding domains within proteins of theSIN3 complex.^[50]^ SIN3 and associated proteins of the complex bind a diverse set of targets to modulate the expression of genes to impact multiple biological processes. In summary, targets of SIN3 regulation represent a wide variety of cellular functions. One report noted the silencing of cell cycle related genes by Sin3 acting with E2F4 and RBP2 in differentiated mouse myoblasts; however, the regulatory effects of SIN3 have generally not been reported to include outright silencing of most target genes, pointing to a modulating role rather than an outright on/off switch.^[51]^

## GLOBAL REGULATION THROUGH SOFT REPRESSION

From a global perspective, Rb and SIN3 share several characteristics, including their preferential association with promoter proximal sequences, their widespread expression, and the diversity of physically and functionally targeted genes. These corepressors differ in that Rb family proteins are regulated by conserved cyclin kinases that affect Rb-E2F association, thus dynamically modulating repression activity, whereas a similar control of SIN3 proteins has not been observed. However, a variety of post-translational modifications do affect SIN3 proteins, and may exert similar regulation.^[52]^ In addition, expression of distinct SIN3 isoforms may adjust SIN3 activity over longer developmental times.

Another common characteristic of these two corepressors, which has been less appreciated, comes from examination of target gene responsiveness to perturbation of Rb or SIN3 proteins. From examination of transcriptomic data, it is apparent that a large portion of their regulons consist of widely active genes, which are subject to fine-tuned regulation. To draw a distinction with a typical silencing action commonly associated with repressors, we call this regulatory activity soft repression, and describe it as the action of repressors/corepressors to modulate or fine-tune gene expression without effectively silencing the promoter. This regulatory action differs from the usual understanding of transcriptional repressors that function as an on/off switch, which for Rb has been demonstrated on cell cycle genes.^[53]^ As discussed below, a closer analysis of these two highly conserved corepressors, Rb and SIN3, indicates that soft repression may be a common, yet underappreciated activity of transcriptional regulators in general.

**FIGURE 2:**
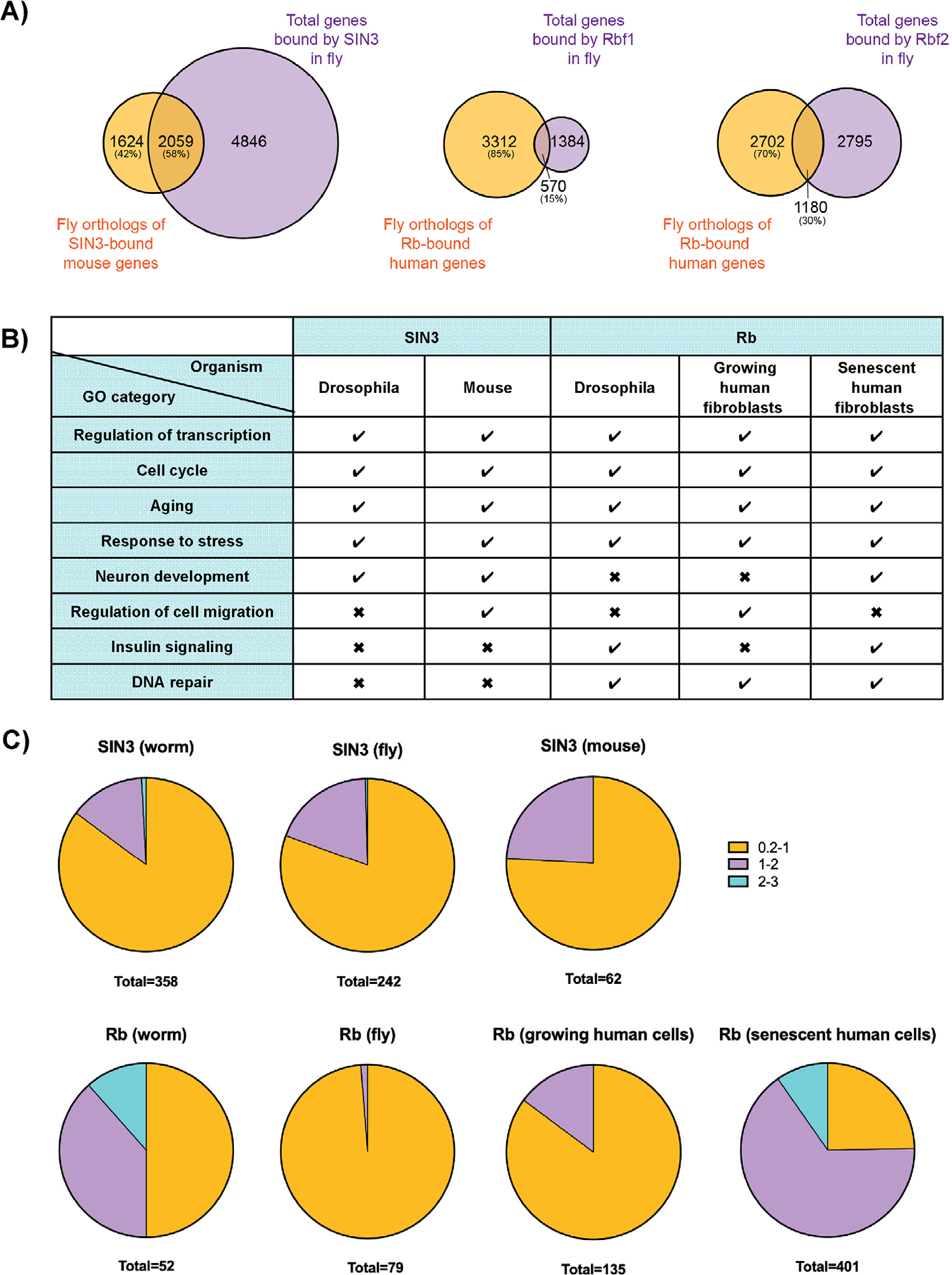
Conserved, direct targets of SIN3 and Rb exhibit soft, but significant repression. (A) SIN3 and Rb bind to a substantial number of the same genes in both the fly and mammalian systems, which indicates conservation of genome-wide binding by these corepressors. To determine this, we used the BioMart data mining tool and analyzed the intersection of fly and mammalian ChIP-seq datasets.^[55]^ Mouse genes for which orthologs can be identified in the fly were overlapped with fly genes bound by SIN3. Similarly, human genes for which orthologs can be identified in the fly were overlapped with fly genes bound by Rb. (B) Chart indicates GO categories misregulated after overexpression or knockdown of SIN3 or Rb in fly and mammalian systems. (C) Pie charts indicate the log_2_-fold change of direct, repressed genes after Rb or SIN3 manipulations in worm, fly, and mammalian models. Totals listed are the number of genes misregulated for each organism and corepressor. Data obtained from^[22,43–45,54,56,Mouawad et al. in prep]^

We performed a comparative examination of published ChIP-seq and transcriptomic data for Rb and SIN3, which supports this new classification of repression mechanisms. We first considered to what extent global regulatory roles of each of these corepressors may be evolutionarily conserved, and compared promoter occupancy of orthologous genes in Drosophila and mammals. Over 50% of all of the genes bound by SIN3A in the mouse that possess a fly ortholog were similarly bound by Sin3A in the fly (Figure 2A). A smaller, but still substantial, fraction of genes bound in human cells by Rb with an ortholog in the fly are bound by Rbf1 and/or Rbf2. The overlap in directly bound targets indicates that both Rb and SIN3 may have conserved roles, although binding does not always predict function. Therefore, to consider functional effects, we combined the ChIP-seq data with available microarray or RNA-seq data from worms, flies, and the mammalian systems (Table 1). We identified functional classes of genes significantly enriched in these lists of direct, repressed targets (Figure 2B). We found that knockdown of *Sin3A* in Drosophila and mouse embryonic stem cells leads to misregulation of genes involved in multiple overlapping processes such as transcriptional regulation, cell cycle, and aging.^[43,44]^ Similarly, in the fly and human cells, regulatory effects of retinoblastoma proteins involved common classes of genes, including transcriptional regulation, cell cycle, and aging, and also included classes not seen to be regulated by SIN3 such as insulin signaling and DNA repair.^[22,54,Mouawad et al. in prep]^

Interestingly, most of the direct, repressed genes showed modest but significant changes in expression after perturbation of Rb or SIN3 (Figure 2C). For example, for both Rbf1 and Sin3A in Drosophila, over 80% of affected genes fell within the lowest category of less than or equal to a two-fold change in expression level (log_2_-fold change, 0.2–1). The knockdown of the Rb worm homolog, *lin-35,* caused a larger range in gene expression changes, 50% having greater than log_2_-fold change of 1. The prevalence of modestly impacted genes suggests that the effects have a biological basis, and that soft repression is observable in these transcriptomic measurements. We note that the bias towards small fold changes may also be the result of incomplete removal of Rb and SIN3 in perturbation experiments, or indirect genetic effects (although we only consider direct ChIP-seq target genes in this analysis). Overall, however, the observed levels of modulation are far from complete silencing of transcription, but do fall well within the levels that are associated with significant biological effects, such as the two-fold changes associated with haploinsufficiency or changes in dosage compensation, both of which can have major consequences. Together, the frequency of soft repression-like effects, along with evidence of evolutionary conservation of physical and functional targeting, suggests that this type of regulation constitutes an important role for both SIN3 and Rb proteins.

Considered on a genome-wide scale, the regulatory action of Rb and SIN3 appears to be largely dedicated to fine-tuning gene activity, although in the case of Rb family proteins, the cell cycle effects have a disproportionate impact on described phenotypes. What evidence would support the important, even predominant, role for soft repression for SIN3 and Rb proteins? First and foremost, SIN3, which apparently is largely restricted to soft repression, is essential in many organisms.^[34,36,37,60,61]^ For Rb, the case is more complex, because standard genetic approaches do not differentiate the impacts of misregulated gene expression involving hard and soft effects. The most compelling evidence comes from Drosophila, where gene duplication and subfunctionalization has apparently partly divided the cell cycle and soft regulatory tasks between Rbf1 and Rbf2. Rbf2—which has many targets within the soft targeting category—is dispensable for development but never lost over longer evolutionary time, likely due to pleiotropic effects.^[16,20]^ Secondly, regulation of a variety of pathways that have been examined in detail show the significant phenotypic consequences of these less than all-or-nothing effects. For Drosophila *Sin3A*, RNAi knockdown upregulates multiple methionine catabolism genes by approximately two-fold.^[13]^ This difference in expression is associated with a change in the level of the key methionine donor SAM, and an increase in H3K4me3, a histone modification linked to gene expression. In the mouse, conditional knockout of *Sin3a* in forebrain excitatory neurons results in a small yet reproducible (20–25%) increase in expression of *Homer1* and cyclin-dependent kinase *Cdk5,* two genes encoding factors involved in memory consolidation, associated with an increase in hippocampal activity.^[62]^

In the case of Rb regulation, one of the most attractive sets of genes for further investigation of soft repression are the over 100 ribosomal protein promoters that are extensively targeted by Rb family members. Ribosomal protein promoters are widely active, thus considered housekeeping in nature, yet respond sensitively to changes in nutrient availability, as well as signals in development and disease.^[63–65]^ The two-fold or less modulation of expression of these genes exerts pleiotropic effects on cellular growth, as evidenced by the *minute* phenotypes caused by haploinsufficiency.^[66]^ While the contribution of Rb proteins to overall expression of these genes still needs to be established, a soft repression level of control is likely to impact cellular growth in a significant way. We propose that this collection of small but reproducible changes contributes to Rb and SIN3 acting as essential global transcriptional regulators that modulate gene expression, rather than fully repress their target genes, to produce measurable biological outcomes.

## THE WHERE AND HOW OF SOFT REPRESSION

Based on gene ontology analysis, targets of soft repression may disproportionately represent housekeeping genes, although this term can be misleading, as it does not mean that the genes are constitutively active in an unregulated manner. To characterize the types of expression patterns observed for Rb and SIN3 target genes, we examined extant gene expression data using modENCODE data accessed through Fly-Base. Focusing on conserved target genes from mammals and flies, we found the majority of these genes to be expressed at all developmental stages, but not in all tissue types. This suggests that these corepressors regulate genes that are widely expressed throughout development, and they potentially modulate their expression in particular contexts. A unifying characteristic of these genes is that despite the presence of the corepressor, they continue to be expressed. To determine whether these target genes are considered stably expressed, we used the tau metric as a computation of gene expression variability.^[67,68]^ A tau value of 0 is given to a gene that is expressed at the same level across the developmental time points and tissue types assayed. A tau value of 1 indicates the gene’s expression is specific to one stage or one tissue type. Using a cutoff of 0.25 as the definition for a “stable gene,” we found that over 50% of conserved Rb or SIN3 targets are considered stable genes throughout development, while only about 25% are stable throughout the tissue types assayed. A hypergeometric test indicates that the stable genes are significantly over-represented within the bound gene sets for both Rb and SIN3. Perhaps a typical promoter that is regulated by soft repression is one that is widely expressed and stable throughout developmental stages.

**FIGURE 3:**
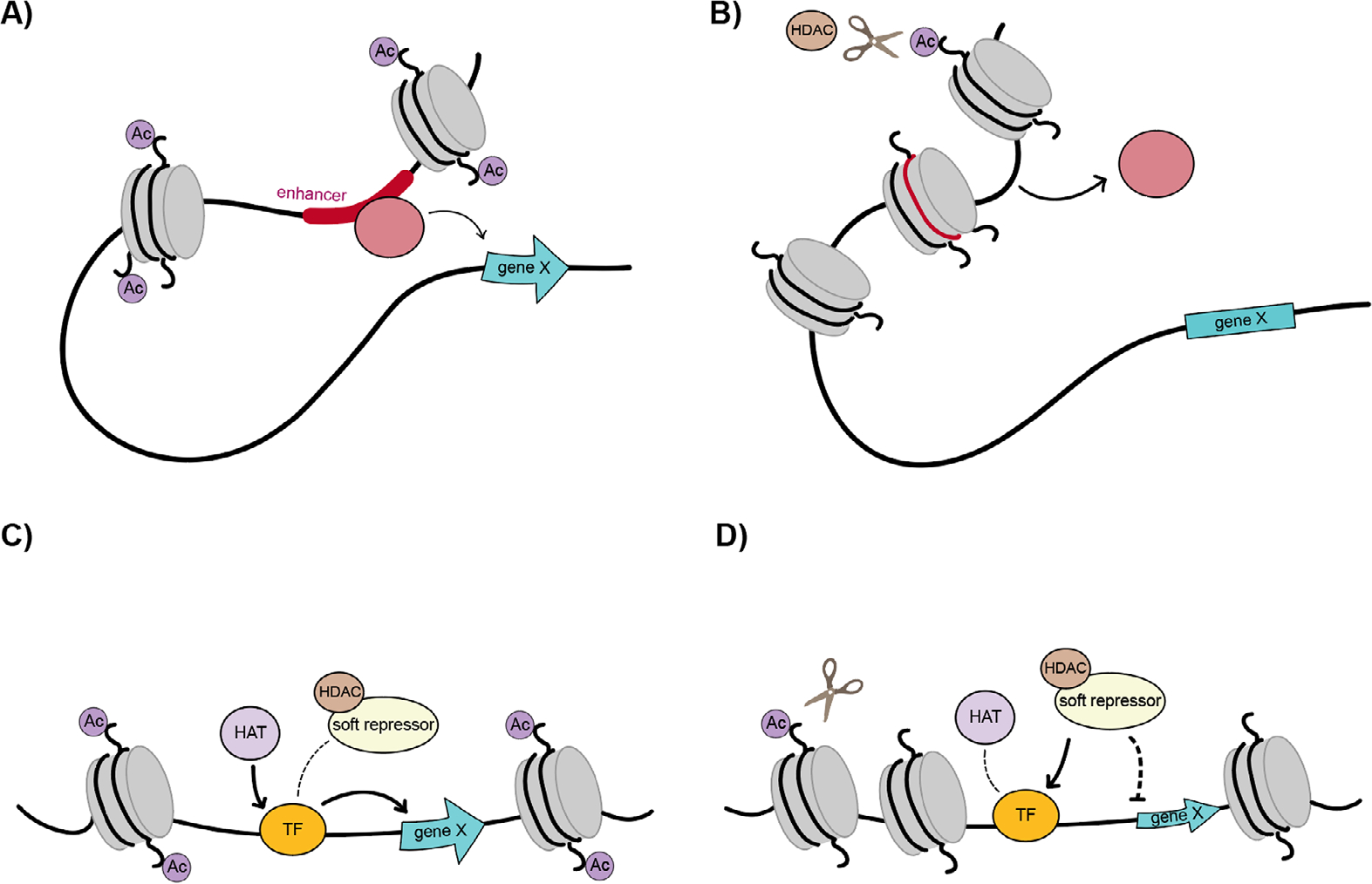
Contrasting models for transcriptional repression: enhancer based hard repression (A, B) and promoter based soft repression (C, D). (A) At enhancers, activators and repressors work in a binary fashion, turning gene expression on and off in response to availability of binding sites and interaction with distal gene promoters. When an activator binds an available enhancer, it can turn on gene expression through chromatin looping. (B) If an enhancer is occluded through nucleosome remodeling and chromatin compaction, activator access is inhibited and the gene is turned off. (C) At promoters, soft repressors can fine-tune expression from the proximity to the transcriptional start site, sometimes through interaction with transcription factors (TF) bound to DNA. There is an interplay between activators and repressors, which compete for DNA recruitment to impact the chromatin environment and modulate gene expression. On the left, a promoter proximal TF interacts with cofactors such as histone acetyltransferases (HAT) to turn on expression of gene X, while on the right, (D) the soft repressor complex, which many times includes a histone deacetylase (HDAC) in the case of Rb and SIN3, is more potent than the activator. The complex deacetylates nearby histone tails and dials down expression of gene X, but does not completely turn it off.

How is it that Rb and SIN3 complexes can be involved in soft repression, when the general types of associated factors - transcription factors and chromatin modifiers such as HDACs - are also involved in more dramatic on/off transcriptional regulation? Our model posits that genes that are fully repressed, perhaps through constitutive and facultative heterochromatin or subject to dominant regulation of distal enhancers, are not subject to control by soft repression (Figure 3A, B). In contrast, the large majority of Rb and SIN3 binding occurs near the transcription start site, rather than at distal enhancers. We speculate that soft repression activity may be exerted strictly from promoter proximal positions, and that specific properties of the promoter region can predispose the regulation to be partial, rather than all-or-nothing (Figure 3C, D). For instance, localization of a repression complex at a promoter may be better suited for partial interference with transcriptional initiation or release, if the biochemical mechanisms invoked (e.g., deacetylation of histone tails, an activity associated with both Rb and SIN3 associated factors) modestly impact nucleosome loading or density. A somewhat inhibited rate of binding or release of RNA polymerase II may then result, possibly changing kinetic constants without blocking essential steps in promoter firing. By contrast, a hard repression effect, such as seen on the *PCNA* promoter, may result from inhibition of the E2F transcriptional activation domain, blocking key interactions with the Mediator or TFIID. It is possible that deployment of similar chromatin modifiers to distal regions may interfere with transcription factor binding and enhancer-promoter looping, resulting in an all-or-nothing effect (Figure 3A, B). Notably, the same biochemical pathways may be involved in hard or soft regulation, and the architecture of the regulatory region would be decisive in dictating the outcome. An alternative and non-exclusive possibility is that soft repression reflects an inherent balance of competitive interactions with the basal machinery, and promoters that feature multiple inputs from other regulatory factors and elements would not be prone to complete silencing (Figure 1B). In summary, it is likely that the context of the regulatory regions in which Rb and SIN3 corepressors operate have a decisive impact on the ability of these factors to play modulating, rather than on/off roles. Further biochemical and molecular biological investigations will be required to elucidate the mechanisms in play. Such studies will likely yield important insights into the dynamic regulation of many constitutively active genes key to metabolism and disease.

## SOFT REPRESSION—A GENERAL PROPERTY OF TRANSCRIPTIONAL CONTROL?

Our studies of soft repression in the context of SIN3 and Rb proteins stemmed from consideration of the many physical targets from genes in shared pathways, which did not exhibit dramatic transcriptional responses, but appeared to explain pleiotropic phenotypes. How general might such regulation be? The physical occupancy of other components of transcriptional regulatory machinery provides clues that continuous repressive activities may be a common feature of many housekeeping promoters. Some of the first genome-wide studies of histone deacetylases revealed that these enzymes, central for repression, are commonly associated with active promoters, in a pattern similar to that of activation-associated histone acetyltransferases.^[69]^ This study did not address the functionality of HDAC at these active loci, although the occupancy positively correlated with gene activity, and the loss of HDAC expression allowed ectopic acetylation of silent transcriptional start sites. One role for HDAC1 at active loci was recently demonstrated to be in the release of paused RNA polymerase II to promote the elongation phase of transcription.^[70]^ As both Rb and SIN3 physically interact with HDAC1, a major mechanism through which these factors exert soft repression could be through regulation of the transition to productive elongation.

An additional aspect of promoter proximal soft repression is that the genes involved may feature multiple regulatory inputs, so that loss of any single regulatory control feature results in a small change in expression. Indeed, many positive and negative regulators of transcription have been found to co-associate with areas of active gene transcription. Using DAM-ID to characterize chromatin association of factors, the van Steensel group performed a genome-wide binding analysis of 53 Drosophila chromatin associated proteins.^[71]^ Certain repressive chromatin marks and factors (e.g., factors of the Polycomb complex) did co-cluster with inactive regions; however, Sin3A and HDAC1 were found to generally colocalize to two subtypes of euchromatin that contain the majority of active genes, and are enriched in binding of components of the general transcription machinery. This study was conducted in cultured cells, where one can expect that most cells are in a similar state of developmental identity. For other types of ChIP-seq analyses of transcriptional regulators that used complex tissues or whole organisms, the co-occurrence of transcriptional repressors with areas of active gene expression may also be a reflection of the heterogeneity of cell types. Thus, to discern whether particular repressors and corepressors may be actively and continuously engaging in modulation of gene expression from specific promoters, it is important to know whether subpopulations of cells are present. Clearly, for widely-active housekeeping-type genes, this concern is of lesser importance. Overall, functional studies - from genetic to genomic - are needed to determine whether the promiscuous association of many factors with certain segments of the genome represents a complex regulatory playing field, or just the noise of binding as countless factors seek their functional targets.

## CONCLUSIONS AND OUTLOOK

From a consideration of SIN3 and Rb, we suggest that many repressors may exert soft repression. There may not be a sharp division between hard and soft repression, but rather a continuum of regulatory action, dependent on promoter architecture, regulatory inputs, and modifications to the corepressors. A focus on soft repression is critical for three reasons: First, this subtle regulation reveals critical direct biological effects, overlooked if stringent quantitative cut-offs are applied. Second, SIN3 and other factors may predominantly act this way, so understanding these factors requires measuring such soft effects. Third, global, systems-level network studies depend on accurate descriptions of genes (nodes) and regulatory interactions (edges), and knowledge of regulation via soft repression will enhance the power of these models.

We see four challenges for progress: First, the magnitude of soft repression makes it hard to spot, since transcriptomic analyses typically focus on more robust effects. Beyond more and deeper sequencing, we need complementary approaches to test the functional importance of potential direct regulation, including high-throughput methods to subtly perturb promoters and repressors and corepressors. Second, pleiotropic effects from misregulation of soft repressors make it difficult to differentiate primary and secondary responses. Approaches that specifically decouple a soft repressor from a target locus in the context of a wild-type cell will be helpful. Third, cell-to-cell variation may complicate distinguishing noise from mild effects. Single cell transcriptomic studies could address this limitation. Finally, a thorough consideration of regulatory variation at the population and species level should be employed to discern possible soft repression effects. Perhaps specific promoter proximal SNPs associated with complex traits impact soft repression. We propose that gene expression research formally consider the impacts of soft repression in myriad settings, to better uncover the basis of gene regulation in development and disease.

## Figures and Tables

**TABLE 1:** Methods used to analyze extent of differential gene expression after genetic manipulations of Rb and SIN3

Reference	Organism	Corepressor manipulation	Technique	DE tool	n samples/condition
[45]	Worm, early embryo	*Sin3* knockout	RNA-seq	DESeq2^[57]^	2–3
[44]	Fly S2 cells	*Sin3A* knockdown	RNA-seq	Cuffdiff^[58]^	3
[43]	Mouse embryonic stem cells	*Sin3A* knockdown	Microarray	Affymetrix procedures and analysis	3
[56]	Worm, larvae	*lin-35* knockdown	RNA-seq	DESeq2^[57]^	2
Mouawad *et al.,* in prep	Fly, larvae	*Rbf1* overexpression	RNA-seq	edgeR^[59]^	3
[22]	Human diploid fibroblasts	*Rb* knockdown	Microarray	Custom statistical model	2

## Data Availability

The data that supports the findings of this study are available in the supplementary material of this article.
